# Effects of an Evidence-Based Falls Risk-Reduction Program on Physical Activity and Falls Efficacy among Oldest-Old Adults

**DOI:** 10.3389/fpubh.2014.00182

**Published:** 2015-04-27

**Authors:** Jinmyoung Cho, Matthew Lee Smith, SangNam Ahn, Keonyeop Kim, Bernard Appiah, Marcia G. Ory

**Affiliations:** ^1^Center for Applied Health Research, Baylor Scott and White Health, Temple, TX, USA; ^2^Department of Health Promotion and Community Health Science, Texas A&M Health Science Center, School of Public Health, College Station, TX, USA; ^3^Department of Health Promotion and Behavior, College of Public Health, The University of Georgia, Athens, GA, USA; ^4^Division of Health Systems Management and Policy, School of Public Health, The University of Memphis, Memphis, TN, USA; ^5^Department of Preventive Medicine, Graduate School of Public Health, Kyungpook National University, Daegu, South Korea; ^6^Department of Public Health Studies, Texas A&M Health Science Center School of Public Health, College Station, TX, USA

**Keywords:** oldest-old adults, falls efficacy, falls risk-reduction program

## Abstract

**Purpose of the study:**

The current study was designed to examine changes in falls efficacy and physical activities among oldest-old and young-old participants in a falls risk-reduction program called a matter of balance/volunteer lay leader model.

**Design and methods:**

An oldest-old group (aged 85 years and older; *n* = 260) and a young-old group (aged between 65 and 84 years old; *n* = 1,139) in Texas with both baseline and post-intervention measures were included. Changes in Falls Efficacy Scale scores and weekly physical activity levels were examined from baseline to post-intervention. Repeated measures analysis of covariance were employed to assess program effects on falls efficacy.

**Results:**

Results showed significant changes in falls efficacy from baseline to post-intervention, as well as a significant interaction effect between time (baseline and post-intervention) and physical activity on falls efficacy.

**Implications:**

Findings from this study imply the effectiveness of evidence-based programs for increasing falls efficacy in oldest-old participants. Future implications for enhancing physical activities and reducing fear of falling for oldest-old adults are discussed.

## Introduction

The population of oldest-old adults – or those 85 years and older – is one of the fastest growing segments of the American population and is estimated to increase from 5.7 million to 19 million by 2050 (1). Despite growth among this population segment, relatively less attention is given to the oldest-old population compared to people aged younger than 85 years old (2). Attention to health status among oldest-old adults is critical because approximately half of those in this age group experience limitations in functioning, which not only impacts their health and independence (3) but also has societal implications on escalating health care utilization and costs (4, 5).

Substantial research has identified functional and behavioral factors associated with loss of independence among the aging population (6, 7). Less physical activity, for example, is increasingly seen as a major contributor to health deterioration and mortality, even among oldest-old adults (8, 9). Lower levels of physical activity contribute to increased number of medical comorbidities in oldest-old populations (10, 11). In addition, anxiety or fear of falling is related to risk for subsequent falls and limited physical activity among older adults (12, 13). For instance, in a sample of adults aged 70 years and older living in a community, over half had fallen at least once during the previous 6 months or restricted their daily activities or both because of a fear of falling (13).

Heterogeneity exists in levels of physical activity among oldest-old adults, despite the lower overall physical activity levels, suggesting the value in identifying modifiable factors associated with higher activity levels. A sense of efficacy, particularly falls efficacy – “the degree of confidence in performing common daily activities without falling” (14) (p. M141) – has been found as a significant factor for physical activity among older adults. Higher levels of efficacy have been related to faster gait speeds (15, 16) as well as lower levels of fear of falling (17); furthermore, physical activity interventions have shown significant positive effects on physical performance related to efficacy (18–20).

### A matter of balance (AMOB) falls risk-reduction program

Previous research indicates that falls risks can be ameliorated, especially through increases in physical activities, which are combined with behavioral strategies to help older adults prevent or manage falls (21–25). Behavioral interventions have been identified to improve falls efficacy (12, 26, 27). A matter of balance (AMOB) is an evidence-based program to reduce falls risk among older adults based on cognitive restructuring methods (28). Established at the Roybal Center for Enhancement of Late-Life Function at Boston University, the original AMOB program was tested through a randomized clinical trial (RCT) (22). The major outcome variables of the program included significant improvements of perceived capacity to manage the risk of falling and confidence in everyday activities without falling. Two primary aims of the AMOB included (a) reducing fear of falling and (b) increasing physical, social, and functional activity (22).

Because the goal of AMOB/volunteer lay leader (VLL) is to build falls efficacy and encourage physical activities, many researchers have examined the effects of AMOB/VLL and found improvement in overall health status, as well as falls efficacy among older adults (29–33). For example, Ory and colleagues (29) found that Texas AMOB/VLL participants showed significant improvements in falls efficacy, physical activity, and normal everyday routines. These results are consistent with other studies that included participants from South Florida and South Carolina (34, 35). Ullmann and colleagues (35) found that South Carolina participants showed greater confidence in managing falls and performing activities of daily living, as well as improvements in functional mobility. In addition to short-term benefits in behavioral outcomes from the program, Smith et al. (36) observed significant yet modest improvements in falls efficacy were maintained 6 months after intervention. Furthermore, improvements in falls efficacy and physical activity have been identified in studies examining the rurality of participants’ residence, participant ethnicity, and the influence of class size and session attendance on health outcomes. Rural residents, Hispanic participants, and participants in smaller size classes with higher attendances showed significant improvements in falls efficacy and physical activity compared to their own counterparts (30, 37, 38).

The extant studies documenting improvements associated with AMOB/VLL typically include a full range of older participants (e.g., all those 65 years and older). Scant research has examined benefits in falls efficacy and physical activities uniquely among oldest-old adults (39). A call has been raised to examine those aged 85 years and older as a separate age group (e.g., a forth age) because of the unique nature and challenges faced by those in this subgroup (40–42). Age-related stereotypes about the benefits of health promotion programs for seniors (43), however, might be a barrier to examining physical activities programs among the oldest-old adults (39). Despite current knowledge of the potential effectiveness of behavioral interventions across the life span (43), few studies have focused specifically on examining the joint influence of falls efficacy and physical activities in the oldest-old population.

**Figure 1:**
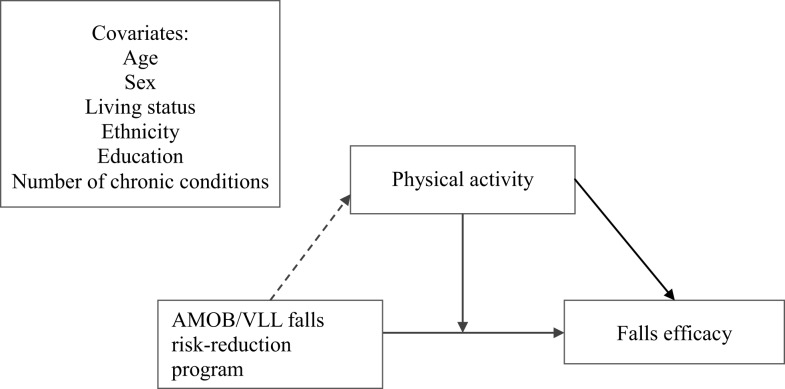
**Conceptual model**.

The purposes of this study were, therefore, to (a) assess the changes in falls efficacy and physical activity from baseline to post-intervention among oldest-old adults and (b) examine the effect of the interaction between improvement of physical activity from baseline to post-intervention on falls efficacy, with a targeted focus on oldest-old participants. A conceptual model for this study is shown in Figure 1. This model depicts the AMOB/VLL falls risk-reduction program as a predictor for changes in physical activity and falls efficacy. In addition, improvement of physical activity acts as a moderator between falls efficacy and falls risk-reduction program.

## Materials and Methods

### Intervention elements

A matter of balance/volunteer lay leader entails a lay leader model and is widely disseminated in the health and aging services sectors (44). The intervention is typically delivered by a pair of trained lay leaders, known as coaches (30, 32, 44). AMOB/VLL was designed to modify fall-related factors, such as behaviors, attitudes, and environmental aspects that increase falling risk among older adults (45). Standardized AMOB/VLL workshops take place at licensed delivery sites and are facilitated by certified coaches to ensure program fidelity (44). As facilitators, these lay leaders use an extensively detailed training manual and two instructional videos (32). The AMOB/VLL intervention consists of eight 2-h sessions either once a week for 8 weeks or twice a week for 4 weeks (30). Early sessions focus on individual behavior and mindsets with an emphasis on decreasing the fear of falling and increasing participants’ confidence to prevent falls; later sessions focus on environmental aspects, so leaders assist participants to change their physical surroundings to reduce risk factors for falling and learn exercises to increase balance and strength (30, 46, 47).

Because the intervention processes focus on building fall-related self-efficacy and setting realistic goals for increasing activity, the intervention includes a variety of components, such as lectures, group discussions, mutual problem solving, role-play activities, exercise training, assertiveness training, and home assignments. A standard definition of a “successful” class completion (i.e., attending five or more of the eight sessions) and an ideal class size (i.e., 8–12 older adults) has been established (38).

### Participants

As noted in our previous research (31, 37), a total of 3,276 participants enrolled in the Texas AMOB/VLL Falls-Prevention Intervention between September 2007 and April 2009 through local area agencies on aging (AAA) and other community-based organizations. Eighteen AAA regions offered 243 classes during that period. The authors obtained Institutional Review Board (IRB) approval at Texas A&M University to analyze secondary data on program participants and the effectiveness of the program.

### Procedures and instruments

The same instruments were used at baseline and after completion of an intervention. A paper-based questionnaire included 28 items. The survey items included four different formats (i.e., Likert-type scales, yes/no, closed response, and open ended). Public health and aging research experts who established a common database for evaluation of program effectiveness in a national consortium of studies helped guide the selection of the measures (48). Participants took approximately 15 min to complete the baseline and post-intervention instruments, respectively.

### Measures

#### Personal characteristics

Six personal characteristic variables were used: age, sex, race/ethnicity, education, living status, and number of chronic conditions. Age was treated as a continuous variable and was based on a participant’s birth date. Sex was scored 1 if the participant is female. Race or ethnicity was scored 0 if the participant is non-Hispanic White, and 1 if non-White. Education was scored 0 if a participant’s highest level of education was less than high school graduation, 1 if graduated from high school, and 2 if more than a high school education. Living status was scored 0 if participants lived alone and 1 if they lived with others. The self-reported number of chronic conditions ranged from zero to seven and was considered as a continuous variable.

#### Falls efficacy scale

Falls efficacy was assessed with the scale developed by Tennstedt et al. (22). The scale consists of five items that measure participants’ perceived ability to manage risk of falls or actual falls (22). Participants were asked to rate the following statements: (1) you can find a way to get up if you fall, (2) you can find a way to reduce falls, (3) you can protect yourself if you fall, (4) you can increase your physical strength, and (5) you can become more steady on your feet. Ratings were used with a four-point Likert scale: 1 = not sure at all, 2 = not very sure, 3 = somewhat sure, and 4 = absolutely sure. Cronbach’s α was 0.87 for the five items of falls efficacy. Scores ranged from 5 to 20 with higher scores indicating higher levels of managing risk of falls. These falls efficacy scores were collected from participants at baseline and post-intervention.

#### Physical activity

Physical activity was measured using one item that asked participants to report the number of days they were physically active in the previous 7 days (i.e., scores could range from 0 to 7 days). Participants were given examples of physical activities (e.g., brisk walking, bicycling, vacuuming, gardening, or anything else that causes one to breathe faster); however, the actual physical activities in which the participant engaged were not independently documented. Physical activity was measured at baseline and post-intervention. Furthermore, the change in the number of days from baseline to post-intervention was assessed. Improvement indicates a greater number of days at post-intervention than baseline; no-improvement indicates a same or less number of days at post-intervention when compared with baseline. Based on the change in number of days from baseline to post-intervention, the authors defined two groups for physical activity: improvement (scored 1) and no-improvement (scored 0).

### Data analysis

Three different analyses were performed. In univariate analyses, frequencies were calculated for personal characteristics, falls efficacy, and physical activity. In bivariate analyses, Pearson’s chi-square tests were conducted to examine the goodness of fit for frequency distributions and the independence between categorical participants’ characteristics (e.g., sex, living status) (49). Multivariate analyses were also performed to obtain adjusted estimates. SAS (ver. 9.2, 2010) Proc Mixed (50) procedures were used when conducting two repeated measures analysis of covariance (ANCOVA) to calculate the adjusted mean changes in falls efficacy scale scores by physical activity groups (i.e., those who showed improvement vs. those with no-improvement). As far as the measurement of physical activity, many previous studies on the program have showed the intervention effects on physical activities (29–33). When we tested a model including physical activity in this study (not shown in this study), this independent variable showed significant effects on falls efficacy in both age groups after controlling for covariates (i.e., slope β = 0.30, *p* < 0.001 for oldest-old group; slope β = 0.28, *p* < 0.001 for young-old group). Assuming significant effects of physical activity on falls efficacy, the physical activity levels were purposively categorized into two groups to see the interaction effect between levels of physical activity (i.e., improvement group vs. no-improvement) and the intervention. In other words, independent variables included time (two time points: baseline and post-intervention) and two levels of physical activity (improvement vs. no-improvement) worked as a moderator. Age, sex, race/ethnicity, education, living status, and number of chronic conditions at baseline were also included as covariates. Many methodological experts of longitudinal studies have advised centering time-varying covariates (51, 52); therefore, we centered age and the number of chronic conditions before conducting advanced analyses. Specifically, we examined whether time (baseline and post-intervention) and two levels of physical activity influence the changes in falls efficacy. In addition, we examined the interaction effect between time (baseline and post-intervention) and physical activity groups (improvement vs. no-improvement) to detect the difference in change of falls efficacy. Covariates and one of the independent variables, time (baseline and post-intervention), were included in the first model. Two levels of physical activity (improvement vs. no-improvement) and interaction term between time (baseline and post-intervention) and physical activity groups were included in the second model.

## Results

### Sample description

**Figure 2:**
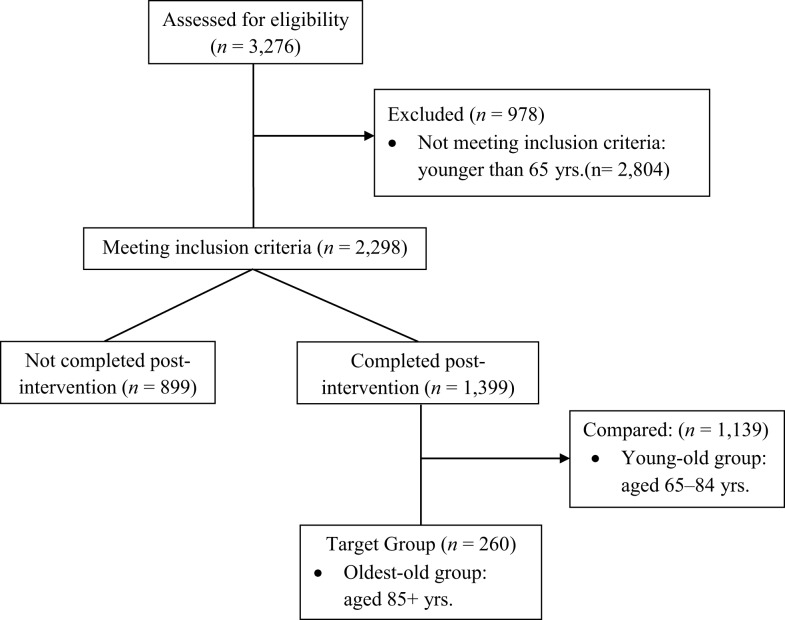
**Diagram for participants inclusion**. No statistical differences found in sex, age, and the number of chronic conditions. Statistical differences found between those who completed and did not complete post-intervention for African-Americans, Hispanics, and those with less than high school education.

As shown in Figure 2, a total of 3,276 participants enrolled in the Texas AMOB/VLL fall risk-reduction program. About 30% of the total participants (*n* = 978) who were younger than 65 years old were excluded. Among those who met our inclusion criteria (*n* = 2,298), 899 participants (39.1%) did not complete post-intervention survey instruments. Only those who completed both baseline and post-intervention assessments (*n* = 1,399) were included in this study. Those aged 85 years and older were categorized into the oldest-old group as a target group for this study (*n* = 260); those aged between 65 and 84 years represent young-old group as a comparison age group (*n* = 1,139).

In addition, we examined characteristics associated with program completion. Although no significant differences were apparent by sex, age, or the number of chronic conditions at baseline (not shown in tables), a significant race/ethnicity difference (*p* < 0.05) was identified between those who completed post-intervention (inclusion group; *n* = 1,399) and those who did not complete post-intervention (exclusion group; *n* = 899). African-American participants represented over 25% and Hispanic participants represented 4.6% among those who did not complete the baseline and post-intervention assessment (exclusion group); African-American participants constituted 17.0% and Hispanic participants constituted 7.9% among those who completed the baseline and post-intervention assessment (inclusion group). Furthermore, a significant education difference (*p* < 0.05) occurred. Those who had less than high school graduation constituted 17.6% among those who completed both baseline and post-intervention (inclusion group), but those who had less than high school graduation constituted 26.4% among the exclusion group.

### Baseline characteristics

Table 1 shows study participants’ characteristics at baseline for those having both baseline and post-assessment data between oldest-old and young-old group. For the oldest-old group, the mean age was 87.84 (SD = 2.84) years old; 76.4% were female, and 70.2% of the group lived alone. The majority of the group (86.5%) was non-Hispanic White, and about half the group (55.5%) had an education above high school. Over 90% of the group (94.6%) attended five or more workshop sessions. The average number of self-reported chronic conditions was 1.64 (SD = 1.14). Participants in the oldest-old group engaged in physical activities on three or more days on average (*M* = 3.55, SD = 2.56). In addition, their average falls efficacy score was 13.58 (SD = 3.92). For the young-old group, the mean age was 76.43 (SD = 5.24) years old; 80% were female, and about half of the comparison group (52.4%) lived alone. The majority of the group (72.5%) was non-Hispanic White, and about half the group (56.8%) had an education above high school. Over 90% of the group (95.9%) attended five and more sessions. The average number of chronic conditions was 1.75 (SD = 1.20). Participants in the young-old group engaged in slightly less physical activities (*M* = 3.46, SD = 2.29). Furthermore, the average falls efficacy score of this group was 14.42 (SD = 3.65).

**Table 1:** **Participant characteristics at baseline**.

	Oldest-old group *(n* = 260)	Young-old group *(n* = 1,139)	*F*/χ^2^
Age^a^	87.84 (± 2.84)	76.43 (± 5.24)	1,156.67***
Sex			1.59
Male	59 (23.6)	216 (20.0)	
Female	191 (76.4)	863 (80.0)	
Living status			26.53***
Living alone	177 (70.2)	575 (52.4)	
Living with one or more others	75 (29.8)	523 (47.6)	
Ethnicity			21.21***
White not Hispanic	212 (86.5)	775 (72.5)	
African-American	21 (8.6)	202 (18.9)	
Hispanic	12 (4.9)	92 (8.6)	
Education levels			0.21
Less than high school	45 (17.6)	196 (17.6)	
High school graduate	69 (27.0)	285 (25.6)	
More than high school	142 (55.5)	632 (56.8)	
Number of sessions attended			
Less than 5 sessions	14 (5.4)	47 (4.1)	0.78
5–8 sessions	246 (94.6)	1,087 (95.9)	
Number of chronic conditions^a^	1.64 (±1.14)	1.75 (±1.20)	1.58
Ave. days of physically active^a^ (0–7)	3.55 (±2.56)	3.46 (±2.29)	0.23
Ave. score of falls efficacy scale^a^ (5–20)	13.58 (±3.92)	14.42 (±3.65)	9.37**

*^a^Means (±SD) reported for continuous variables*.

**p < 0.01, ***p < 0.001.

### Change in falls efficacy

Table 2 presents the results of repeated measures ANCOVA in the oldest-old group and the young-old group. Two models were compared for each group in Table 2. For the oldest-old group, time was statistically significant for change of falls efficacy from baseline to post-intervention in Model 1. In other words, the mean scores of falls efficacy scores significantly increased from baseline to post-intervention (slope: β = 1.98, *p* < 0.001). In addition, improvement of days of physical activities and the interaction term between time (baseline and post-intervention) and physical activities were included in Model 2. Both physical improvement and the interaction term were significant (slopes: β = 1.32, *p* < 0.05, β = 1.43, *p* < 0.05, respectively). These results indicate that the mean score of falls efficacy in improvement group was higher than in the no-improvement group and mean scores of falls efficacy in both improvement group and no-improvement group at baseline were different from those at post-intervention.

**Table 2:** **Models for changes in falls efficacy among oldest-old group and young-old group**.

Predictors	Oldest-old group (*n* = 190)		Young-old group (*n* = 1,015)	
	Model 1	Model 2	Model 1	Model 2
Intercept	15.50	14.98	11.96	12.40
Covariates				
Age	−0.17 (0.07)*	−0.16 (0.07)*	−0.01 (0.01)***	−0.07 (0.01)***
Sex (female = 1)	−0.99 (0.51)	−0.93 (0.51)	−0.62 (0.22)**	−0.57 (0.51)*
Living status (living alone = 0)	−0.68 (0.48)	−0.63 (0.49)	0.16 (0.17)	0.19 (0.18)
Ethnicity (White not Hispanic = 1)	−0.78 (0.69)	−0.66 (0.69)	0.24 (0.22)	0.08 (0.24)
Education (less than HS = 1)	0.33 (0.29)	0.29 (0.29)	0.28 (0.12)*	0.34 (0.14)*
Number of chronic condition	−0.25 (0.19)	−0.22 (0.19)	−0.52 (0.07)***	−0.52 (0.08)***
Time (baseline = 0)	1.98 (0.30)***	1.33 (0.39)***	2.03 (0.12)***	1.71 (0.16)***
Improvement of physically active (Improved = 1)		1.32 (0.52)*		1.05 (0.42)*
Time*improvement of physically active		1.43 (0.58)*		0.73 (0.25)**
AIC (Akaike’s information criteria)	1818.3	1814.6	9640.2	8359.1

*Figures shown in the table are metric coefficients and standard errors (in parentheses)*.

*p < 0.05, **p < 0.01, ***p < 0.001.

Similar results of changes in falls efficacy were found in the young-old group. Time was statistically significant for change of falls efficacy from baseline to post-intervention in Model 1 (slope: β = 2.03, *p* < 0.001); both physical improvement and the interaction term were statistically significant (slopes: β = 1.05, *p* < 0.05, β = 0.73, *p* < 0.01, respectively) in Model 2.

### Relationship between improvement of physical activities and falls efficacy

**Figure 3:**
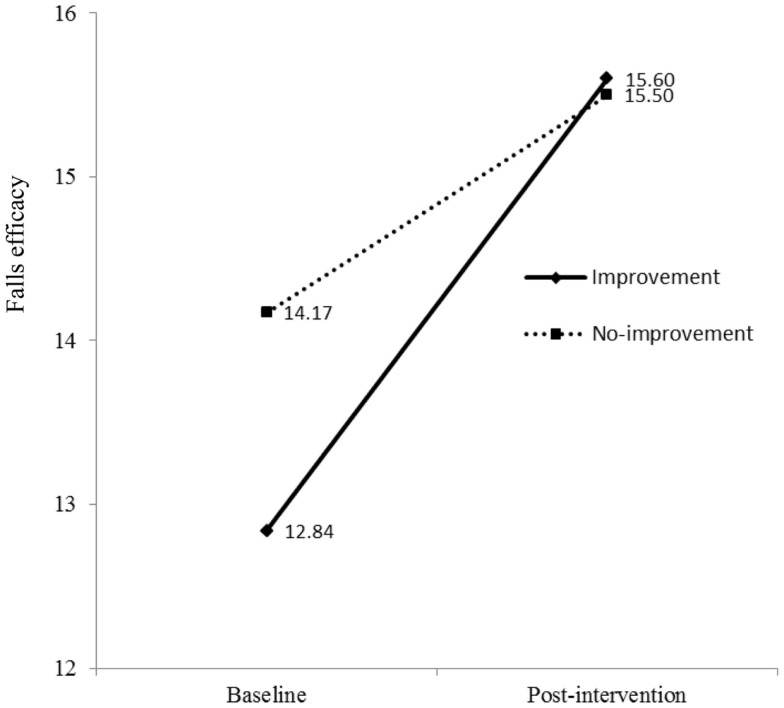
**Falls efficacy at baseline and post-intervention by physical activity groups (improvement vs. no-improvement) in oldest-old group**.

**Figure 4:**
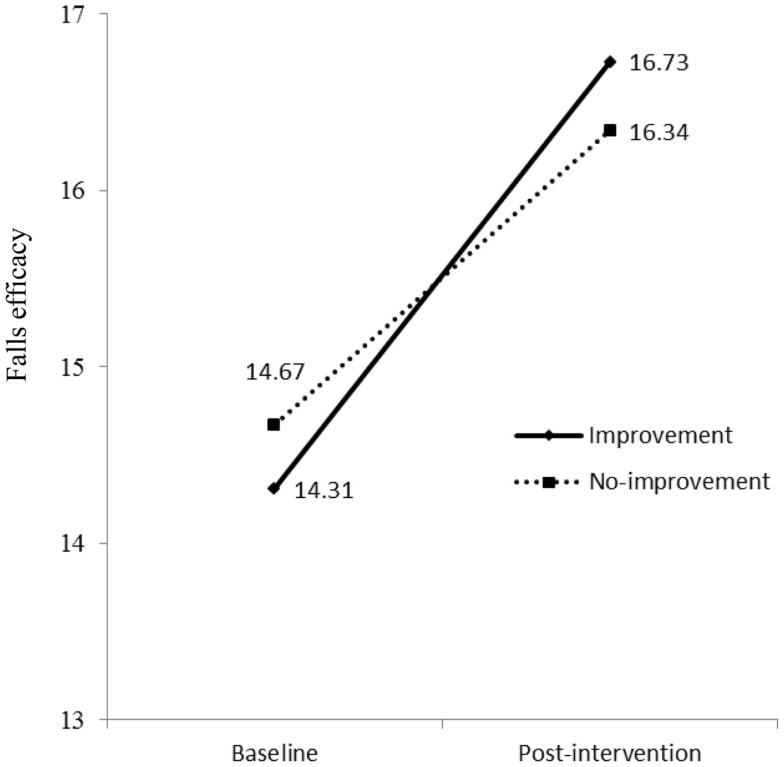
**Falls efficacy at baseline and post-intervention by physical activity groups (improvement vs. no-improvement) in young-old group**.

As shown in Model 2 (Table 2), the interaction term between time (baseline and post-intervention) and physical activity had significant effects on falls efficacy in both oldest-old and young-old groups. This indicates mean scores of falls efficacy in both improvement group and no-improvement group at baseline were different from those at post-intervention. To examine interactions, methodologists have advised plotting the figure (53). As shown in Figure 3 for oldest-old group, the improvement group in physical activity had lower score of falls efficacy at baseline than the no-improvement group, but after they participated in the AMOB/VLL program, their falls efficacy score improved greater than the participants in the no-improvement group. In other words, the improvement in falls efficacy was associated with increased physical activity as well as program participation among oldest-old participants. The young-old group also showed same trends; the improvement group in physical activity had lower score of falls efficacy at baseline, but their score improved greater than the participants in the no-improvement group (Figure 4).

## Discussion

Many previous studies have assessed falls efficacy and physical activities among participants in the AMOB/VLL program and shown the effectiveness of the program. Most studies, however, did not differentiate oldest-old participants from general old adults. Specification of age group may contribute to a closer look at the effectiveness of evidence-based program in falls efficacy and physical activities. The aim of this study was to examine physical and psychological benefits among oldest-old adults enrolled in the Texas AMOB/VLL falls risk-reduction program. First, this study contributes to understanding of falls efficacy among oldest-old adults by extending the evidence base of the AMOB/VLL falls risk-reduction program. Our findings confirm the increased falls efficacy among oldest-old adults in Texas similar to that reported previously for the general population of older adults (29, 31, 38, 44) From baseline to post-intervention, oldest-old participants who enrolled in the AMOB/VLL intervention showed significant improvement in falls efficacy as shown in the young-old group. This finding indicates that entry into the program may have a significant effect on changes in confidence of managing falls-related risks from baseline to post-intervention. A few studies showed that the effect of psychological variables is attenuated for those over 75 years old (54, 55). Our results, however, indicate that older adults, especially those aged 85 years and older, can improve their own self-beliefs related to risks of falling through intervention (56, 57).

Second, this study suggests a mechanism for overcoming psychological barriers. Our results suggest that increased physical activities contributed to improving falls efficacy among oldest-old adults enrolled in an evidence-based falls risk-reduction program. From baseline to post-intervention, as noted above, participants showed significant improvement in falls efficacy; however, 44% of participants who enrolled in the intervention showed significant improvement in days of physical activities in the improvement group while 56% of participants indicated decline or same days of physical activities in the no-improvement group. At baseline, the falls efficacy scores of the improvement group (*M* = 12.84, SD = 4.78) were lower than those of the no-improvement group (*M* = 14.17, SD = 4.35). There was a significant difference between the two falls efficacy scores, *t*(306) = 2.57, *p* < 0.01. This indicates that the AMOB/VLL intervention contributed to differential improvement in the falls efficacy between the two groups. At post-interventions, falls efficacy scores of the improvement group and no-improvement group were 15.60 (SD = 4.71) and 15.50 (SD = 4.18), respectively. There was no significant difference between the two falls efficacy scores at post-intervention, *t*(305) = 0.198, *p* = 0.579.

This finding provides another significant benefit of evidence-based programs in improving the quality of life among oldest-old population. Most studies related to the effectiveness or benefits of evidence-based program have focused on separate health-related outcomes, such as health behaviors, self-efficacy, or falling or injury rates (29, 58, 59). The results of this study, however, provide critical evidence suggesting that the AMOB/VLL program can positively affect psychological beliefs (i.e., falls efficacy), as well as physical activities among oldest-old participants at the same time. One possible explanation of the synergy/doubled/combined effect of physical and psychological improvement from the falls risk-reduction program may be that oldest-old adults had more barriers for physical activities than younger counterparts. Through a systemic review of physical activity in oldest-old adults, Baert and colleagues (60) have reported many different types of barriers, such as physical impairment (61), weakness of physical strength (62), being too tired (59), fear of injury or pain (63), or the belief that older people cannot change (64). Our oldest-old participants enrolled in the AMOB/VLL intervention may, however, overcome those barriers. In particular, the group that improved their physical activities may enhance ability or strategy of prevention of falls risks and this, in turn, contribute to improve falls efficacy.

### Limitations

Some limitations were related to this study, despite noteworthy findings. First, the study variables collected at baseline and post-intervention were self-reported. We should consider the possibility of recall bias because participants were asked to recall occurrences within the previous week or month (31). Second, the participants in this study were recruited from only one geographic region of the United States (i.e., Texas). Participants from more demographically diverse states of United States or other countries might demonstrate different patterns in the change in falls efficacy. More studies from other states and in diverse settings could contribute to generalization of the results. Third, participants were not randomly assigned into the intervention, nor were a true comparison group included in the study design (i.e., older adults who did not receive the AMOB/VLL intervention). With translational research studies, the main objective is to replicate outcomes previously obtained in more controlled intervention designs across different groups. Hence, such translational studies are often not designed as RCTs (65); nevertheless, our use of a one group design in this study limits our ability to definitively confirm the presence of significant intervention effects between baseline and post-intervention on falls efficacy and physical activity. As such, we recommended that future studies include both intervention and comparison groups to detect true intervention effects (e.g., RCT by specified age groups). Admittedly, self-selection bias may be another limitation for this study because participants chose to enroll in the AMOB/VLL program. However, our findings are similar to those reported in other studies in which no treatment comparison group was used (22, 44).

Fourth, the single item used to measure physical activity asked participants to report the number of days they were physically active in the previous 7 days. We were limited in our ability to perform more complex analyses with this variable or weight specific physical activities. In addition, because this variable simply asks the number of days physically active, not the number of minutes, the ability to detect change is less because the item is not very sensitive to change. Thus, we elected to measure changes in physical activity from baseline to post-intervention as “improved/not improved.” We acknowledge that this decision may be influenced by a potential ceiling effect among those who were physically active upon entering the program. This may have accounted for fewer significant improvements in physical activity to be observed at post-intervention. If a more specified scale (e.g., measuring duration such as minutes per activity) or more specific items related to falling (exercise vs. daily living) were used, the effectiveness of fall-reduction programs may become more pronounced. Fifth, 899 participants who did not complete post-intervention assessments were excluded from study analyses. The target group of this study was participants aged 85 years and older; thus, we believe a majority of missing data from participants aged from 65 to 84 years did not strongly impact our intervention findings. Nevertheless, strategies are needed to raise participant retention and assessment response rate, which can reduce a selection bias in future implementation efforts. Sixth, Figures 3 and 4 show that intervention effects influenced changes in falls efficacy levels differently for physical activity groups, regardless of age groups. Because baseline levels of falls efficacy were substantially lower in the improvement group compared to the no-improvement group, the effect on improvement group participants would be expected to be larger than the no-improvement group participants. Again, a ceiling effect may account for the less change in falls efficacy for the no-improvement group relative to the improvement group. Levels of falls efficacy at post-intervention were similar in both groups. Because, regardless of age, participants in this study showed significant improvements after the intervention, we acknowledge there may be other extraneous effects that were not captured in this study. Future researchers may elect to collect a more encompassing set of measures to assess the complex factors associated with falls efficacy improvement among participants. Finally, only a short-term assessment of this intervention program was conducted (e.g., at 8 weeks post-intervention initiation). The study outcomes may be more robust if participants were studied for 6 months or 1 year (66).

### Implications for research

The findings from this study have considerable implications for future research on aging studies. Most notably, the inclusion of the young-old group in this study emphasizes the magnitude of intervention benefits for the oldest-old population. Although it is expected that younger seniors may benefit from the intervention more than their older counterparts, findings of this study indicate both groups’ improvement in physical activity was associated with improvements in falls efficacy. Moreover, oldest-old participants showed larger rate of improvement when compared to the younger-old participants. Future studies should focus on participants aged 85 years and older to examine what characteristics are associated with the effectiveness of evidence-based programs, such as AMOB/VLL. Detailed examination of whether physical activities from the AMOB/VLL could influence cognitive function/mood, remove barriers for physical activities, or improve those with specific conditions, such as dementia, are foci for future research. This study examined an interaction effect between physical activity and time (from baseline to post-intervention) on falls efficacy among oldest-old adults. As a couple of differences between those included and excluded were identified (i.e., education and ethnicity) and additional interaction effects were not investigated in this study, we acknowledge there may have been other factors that influenced program participation and falls efficacy among these participants. More specifically, future studies should explore confounding effects among participant samples with diverse racial/ethnic backgrounds (e.g., African-American, Hispanic) and differing education levels (e.g., 17.6% did not complete high school) to assess their influences on intervention effects.

### Implications for practitioners and policy makers

The results of this study suggest that more practical and policy applications are needed, especially for oldest-old population. Although the oldest-old group (i.e., over 85 years old) will form a large proportion of global population in the next couple of decades (1, 39), few studies have been conducted on the effectiveness of evidence-based programs for oldest-old population compared to younger groups (i.e., younger than 85 years old) (67). In contrast to misconceptions and age-related stereotypes (39), the results of this study suggest that systematic strategies must be employed to develop falls risk-reduction programs for oldest-old adults. We recommend that falls risk-reduction programs be developed or modified, specifically targeting different age groups (e.g., younger than 85 years old vs. 85 years old and over) and different levels of physical activities. This may allow oldest-old adults to gain a more powerful intervention effect and to enhance their physical activities and falls prevention and, in turn, may contribute to reducing medical expenses for falls; furthermore, staff from nursing homes or senior centers as well as health professionals could be trained to develop appropriate ways to make environments more physical activity friendly for oldest-old residents in long-term care facilities.

To summarize, findings from the present study are unique in that they show simultaneous physical and psychological benefits of AMOB/VLL among oldest-old participants. This study re-emphasizes the critical effectiveness of an evidence-based fall risk-reduction program on oldest-old participants by increasing their levels of physical activity and falls efficacy. Identifying characteristics of oldest-old participants who benefit from this intervention has the potential to enhance its effectiveness and inform the development of systematic strategies to encourage enrollment and participation among oldest-old adults.

## Conflict of Interest Statement

The authors declare that the research was conducted in the absence of any commercial or financial relationships that could be construed as a potential conflict of interest.

This paper is included in the Research Topic, “Evidence-Based Programming for Older Adults.” This Research Topic received partial funding from multiple government and private organizations/agencies; however, the views, findings, and conclusions in these articles are those of the authors and do not necessarily represent the official position of these organizations/agencies. All papers published in the Research Topic received peer review from members of the Frontiers in Public Health (Public Health Education and Promotion section) panel of Review Editors. Because this Research Topic represents work closely associated with a nationwide evidence-based movement in the US, many of the authors and/or Review Editors may have worked together previously in some fashion. Review Editors were purposively selected based on their expertise with evaluation and/or evidence-based programming for older adults. Review Editors were independent of named authors on any given article published in this volume.
